# Serotonin mediation of early memory formation via 5-HT_2B_ receptor-induced glycogenolysis in the day-old chick

**DOI:** 10.3389/fphar.2014.00054

**Published:** 2014-04-01

**Authors:** Marie E. Gibbs, Leif Hertz

**Affiliations:** ^1^Drug Discovery Biology, Monash Institute of Pharmaceutical Sciences, Monash UniversityParkville, VIC, Australia; ^2^Department of Clinical Pharmacology, China Medical UniversityShenyang, China

**Keywords:** serotonin, glycogenolysis, day-old chick, memory formation, astrocytes

## Abstract

Investigation of the effects of serotonin on memory formation in the chick revealed an action on at least two 5-HT receptors. Serotonin injected intracerebrally produced a biphasic effect on memory consolidation with enhancement at low doses and inhibition at higher doses. The non-selective 5-HT receptor antagonist methiothepin and the selective 5-HT_2B/C_ receptor antagonist SB221284 both inhibited memory, suggesting actions of serotonin on at least two different receptor subtypes. The 5-HT_2B/C_ and astrocyte-specific 5-HT receptor agonist, fluoxetine and paroxetine, enhanced memory and the effect was attributed to glycogenolysis. Inhibition of glycogenolysis with a low dose of DAB (1,4-dideoxy-1,4-imino-D-arabinitol) prevented both serotonin and fluoxetine from enhancing memory during short-term memory but not during intermediate memory. The role of serotonin on the 5-HT_2B/C_ receptor appears to involve glycogen breakdown in astrocytes during short-term memory, whereas other published evidence attributes the second period of glycogenolysis to noradrenaline.

## INTRODUCTION

The importance of glycogenolysis for successful learning has been overwhelmingly demonstrated (O’Dowd et al., 1994; Hertz et al., 2003, 2013; Gibbs et al., 2006a, 2007; Suzuki et al., 2011; Gold and Korol, 2012; Duran et al., 2013). During one-trial aversive learning in the day-old chick, the exact timing of not one, but three periods of glycogenolysis is well established, i.e., 5, 30, and 55 min post-training (Hertz and Gibbs, 2009). Our previous experiments focused on the glycogenolytic and glycogen synthesis-promoting effect of noradrenaline, via β_2_- and α_2_-adrenergic receptors respectively (Gibbs et al., 2008a; Gibbs and Hutchinson, 2012). It indicated β_2_-adrenergic receptor stimulation as the signal leading to the second glycogenolytic period, but also that noradrenaline was not implicated in starting the first. Several other transmitters, including serotonin, activate glycogenolysis in brain tissue and in cultured astrocytes (Quach et al., 1982; Cambray-Deakin et al., 1988; Magistretti, 1988; Chen et al., 1995; Kong et al., 2002; Darvesh and Gudelsky, 2003). This study investigates serotonin in detail, and it eventually focuses on the role of the 5-HT_2B_ receptor. However, unlike noradrenaline, which also stimulates glycogen synthesis (Hertz et al., 2007; Hutchinson et al., 2008, 2011; Gibbs and Hutchinson, 2012), 5-HT is not known to stimulate glycogen synthesis in brain.

As in our previous studies, this report uses avoidance training in the day-old chick, a precocious animal. Before or during the training drugs are injected into the intermediate medial mesopallium (IMM), an avian equivalent of the mammalian cerebral cortex. This learning task in the premature chick brain shares many characteristics with inhibitory learning in the adult mammalian hippocampus (Izquierdo and Medina, 1997). During a 10 s learning period the chicks learn to associate a red bead with aversive taste, and consolidation of this memory is indicated by unwillingness to peck at untainted red beads, while freely pecking at corresponding blue beads. Time of injection-dependent inhibition of learning by metabolic inhibitors and restoration of memory by specific substrates at precisely determined times allow determination of specific metabolic events and their neuronal or astrocytic localization (Gibbs et al., 2008a).

Following inhibition of glycogenolysis during either of the two first glycogenolytic periods, memory disappears after 30 min and inhibition of the third glycogenolytic period leads to amnesia after 70 min. The first period of glycogenolysis almost immediately after training, provides the substrate for formation of transmitter glutamate (Hertz et al., 2003; Gibbs et al., 2007), an astrocytic process (Hertz, 2011). The transmitter stimulating glycogenolysis at this time cannot be noradrenaline, since an antagonist of the β_2_-adrenergic drug, propranolol, administered prior to the *first* period of glycogenolysis does not inhibit memory (Gibbs and Summers, 2002). The next glycogenolytic period around 30 min is triggered by noradrenaline (Gibbs and Summers, 2002). Like the first glycogenolytic period it also occurs immediately before a known release of transmitter glutamate (Daisley et al., 1998). It is likely, although not proven that glycogen again serves as a glutamate precursor. However, unlike the first period the use of glycogen is not reflected by a significant decrease in its level (O’Dowd et al., 1994), probably reflecting that noradrenaline stimulates both glycogenolysis and glycogen synthesis. The fate of pyruvate/lactate derived from glycogen during the third glycogenolytic period 55 min post-training is unknown, and inhibition of glycogenolysis causes memory to disappear around the onset of long-term protein-synthesis-dependent memory (Gibbs and Ng, 1984). In contrast to the first two glycogenolytic periods intracerebral injection of the glutamate precursor glutamine does not rescue memory after the third glycogenolytic period (Gibbs et al., 2008a).

Our previous studies have suggested that serotonin has both memory-enhancing and memory-inhibitory effects on learning in day-old chicks (Gibbs et al., 1987; Hertz and Gibbs, 2009). The prime purpose of the present study has been to determine which 5-HT receptor is responsible for the memory-enhancing effect of serotonin and to investigate whether it may play an essential role in triggering the first glycogenolytic response during learning in day-old chicks. During the course of this investigation information was also gathered regarding the ability of *high* concentrations of serotonin to inhibit memory. In this study serotonin itself, different serotonin antagonists, and the subtype-specific 5-HT_2B_ receptor agonists, fluoxetine, and paroxetine were used.

There are seven 5-HT receptor families: 5-HT_1_ – 5-HT_7_ (Uphouse, 1997). With the exception of the 5-HT_3_ receptor, a ligand-gated cation channel, they are all G protein-coupled. 5-HT_1_ (Uphouse, 1997) and 5-HT_5_ (Volk et al., 2010) receptors are G_i_/G_o_-coupled and their activation decreases adenylate cyclase activity. However, blockade of presynaptic 5-HT_1_ receptors also enhances 5-HT_2_-mediated activities (Fox et al., 2010). Members of the 5-HT_2_ family (A, B, and C) are G_q_/G_11_-coupled and signal via phospholipase C (PLC) and the phosphatidylinositide second messenger system. This includes a rise in free cytosolic Ca^2^^+^ ([Ca^2^^+^]_I_ which is of major importance because a [Ca^2^^+^]_I_ increase is indispensable for glycogenolysis, not only in muscle (Ozawa, 1972, 2011) but also in brain (Ververken et al., 1982) and in cultured astrocytes (Xu et al., 2014). 5-HT_4_, 5-HT_6_, and 5-HT_7_ receptors are G_s_-coupled and linked to activation of the adenylate cyclase system, generating c-AMP (Uphouse, 1997). However, in contrast to prevailing concepts, c-AMP on its own cannot elicit glycogenolysis (Ozawa, 1972, 2011; Ververken et al., 1982), whereas it can increase the glycogenolytic effect during increases in [Ca^2^^+^]_i_ (Ozawa, 1972).

The two “serotonin-specific reuptake inhibitors” (SSRIs), fluoxetine, and paroxetine are specific 5-HT_2B_ agonists in cultured astrocytes (Li et al., 2008; Zhang et al., 2010; Hertz et al., 2012). Diaz et al. (2012) have confirmed that fluoxetine administration stimulates the 5-HT_2B_ receptor, probably also on raphe neurons, which were found to express 5-HT_2B_ receptors. This might be the reason for serotonin reuptake inhibition, which the authors still regarded as the mechanism for the functional effects of SSRI’s. However, this is impossible in the cultured astrocytes, which express no serotonin transporter (SERT; Kong et al., 2002). Nevertheless, in cultured cells both fluoxetine and paroxetine are subtype-specific agonists of the astrocytic, but not the neuronal, 5-HT_2B_ receptor, with a moderately high, almost similar, acute affinity, i.e., a K_i_ value for displacement of serotonin of 70 nM (Hertz et al., 2012). This is a pronounced difference from their affinity for all other 5-HT receptors, and since the 5-HT_2B_ receptor was unknown at the time fluoxetine came on the market, the conclusion that it had negligible receptor affinity was correct at that time. The almost similar affinities of fluoxetine and paroxetine for the 5-HT_2B_ receptor (Zhang et al., 2010) occur in spite of the fact that the affinities of these two drugs for the 5-HT transporter (SERT) and for the 5-HT_2C_ receptor are widely different (Wong and Bymaster, 1995). These drugs are therefore able to distinguish between the two 5-HT_2_ receptor subtypes and between effects on astrocytes and neurons. Provided they are approximately equipotent, the possibility that they act by inhibiting the SERT is eliminated.

Different 5-HT receptor families and their subtypes are more or less selectively inhibited by a large variety of drugs. In the present study we tested methiothepin, an inhibitor of 5-HT_1_, 5-HT_2_, 5-HT_5_, 5-HT_6_, and 5-HT_7_ receptors, although with different potencies (Misane and Ogren, 2000), and the more specific 5-HT_2B/C_ receptor antagonist, SB221284 (Bromidge et al., 1998).

Two different learning paradigms were used: (i) strong learning established by pecking on a bead tainted with the taste aversant anthranilate in undiluted form (100% anthranilate), a procedure which leads to spontaneous consolidation into long-term memory; and (ii) weak learning after similar exposure but to 20% anthranilate, which initially also leads to memory retention, but where the memory trace normally fades after 30 min, but can be rescued by specific interventions.

## MATERIALS AND METHODS

### ANIMALS

Between 120 and 180 day-old cockerels from an egg-laying hybrid strain (New Hampshire, Rhode Island Red, White Leghorn and Black Australorp) were delivered from a local hatchery (Wagner’s Poultry, Coldstream, VIC, Australia) on the morning of each experiment. Details of housing and experimental conditions are described in detail by Gibbs and Summers (2002). Chicks were divided into groups of 16–20.

### DRUGS AND THEIR ADMINISTRATION

Drugs: (i) The universal 5-HT receptor agonist, 5-hydroxytryptamine hydrochloride, acting potently at 5-HT_2A_ and 5-HT_2B_ receptors (Doyle et al., 1986; Misane and Ogren, 2000; Li et al., 2010), as well as at 5-HT_6_ and 5-HT_7_ receptors but less potently at 5-HT_1_ receptors (Granados-Soto et al., 2010); 5-HT_3_ (Chetty et al., 2006), 5-HT_4_ (Prins et al., 2000), 5-HT_5_ (Thomas et al., 2000; Nelson, 2004), and 5-HT_6_ (Monsma et al., 1993) receptors; (ii) fluoxetine hydrochloride and paroxetine hydrochloride (from Sigma-Aldrich, St. Louis, MO, USA), subtype-specific, almost equipotent agonists at the astrocytic 5-HT_2B_ receptor; (iii) methiothepin maleate (1-[10,11-dihydro-8-(methyl-thio)dibenzo(*Z*)[*b,f*]thiepin-10-yl]-4-methylpiperazine maleate), from Research Biochemicals, Inc, a non-specific antagonist of all 5-HT receptors, except 5-HT_3_ and 5-HT_4_ (Misane and Ogren, 2000), which has high affinity for 5-HT_2A_ (Briejer et al., 1997) and 5-HT_2B_ receptors (Glusa and Pertz, 2000), 5-HT_5_ receptors (Thomas et al., 2000), 5-HT_6_ receptors (Misane and Ogren, 2000) and 5-HT_7_ receptors (Jänichen et al., 2005), but lower affinity for at least some of the 5-HT_1_ receptors (Misane and Ogren, 2000); (iv) the selective 5-HT_2B/C_ receptor antagonist SB221284 (2,3-dihydro-5-(methylthio)-*N*-3-pyridinyl-6-(trifluoromethyl)-1*H*-indole-1-carboxamide), from Tocris Cookson Ltd. UK. All drugs were prepared in sterile physiological saline (154 mM NaCl). Central injections of 5 or 10 μl were made into the IMM of each hemisphere using a Hamilton Repeating Dispenser syringe fitted with a 27-gage needle with a stop controlling the depth to 3.5 mm. Control chicks were injected with saline; (v) the glycogenolysis inhibitor DAB (1,4-dideoxy-1,4-imino-D-arabinitol; Sigma-Aldrich, St.Louis, MO, USA) was administered by subcutaneous injections of 100 μl.

All experimental procedures were in accordance with the guidelines approved by the Monash University Animal Ethics Committee and comply with the 1997 Australian Code of Practice for the care and Use of Animals for Scientific Purposes. All efforts were made to minimize both the suffering and the number of animals used. Chicks were killed at the completion of each experiment by CO_2_ inhalation.

### EXPERIMENTAL PROCEDURE

The procedures have been described in detail elsewhere (Gibbs and Summers, 2002; Gibbs et al., 2008a). Chicks were familiarized with the introduction of small shiny beads and subsequently, with colored red and blue beads, into their cage prior to the training trial. The beads were dipped in water. Chicks peck equally at both colors of the bead. For the training trial, an identical red bead was dipped in either 100% methyl anthranilate (strongly reinforced training) or 20% anthranilate diluted in alcohol (weakly reinforced training) and presented to each pair of chicks for 10 s. On the retention test, the chicks were presented with a clean dry red bead, followed by a clean dry blue bead 2.5 min later, and allowed 10 s to peck at each bead. The number of pecks at each bead and the latency to the first peck in all trials with the colored beads was recorded on a hand held logger, later decoded by computer. Drugs were administered, and testing carried out, at predetermined times relative to the learning trial. Memory is retained after both weakly reinforced and strongly reinforced training, in the former for only 30 min (unless reinforced pharmacologically), compared to least 24 h after the latter (unless inhibited pharmacologically), although with two brief declines in intensity, normally at 15 and 55 min. Memory retention is indicated by many fewer pecks on the blue bead compared to the red one.

At the completion of each experiment, data were retrieved from the computer and results calculated. At this point the pecking data from chicks failing to peck at the red bead during training or at the blue bead on test were excluded from further analysis. Memory was indexed by a discrimination ratio (DR), defined as the ratio of the number of pecks at the blue bead to the total number of pecks at the red and the blue bead. All statistical tests were one-way ANOVA unless otherwise specified. The one-way ANOVA were followed by *post hoc* analysis using Dunnett’s test, with P set at 0.05.

### EXPERIMENTAL PROTOCOLS

#### Dose-response relationships

Dose-response studies were performed with injection immediately after training and memory tested 120 min later to establish enhancement of memory after weakly reinforced training (on 20% methyl anthranilate) or inhibition of memory after strongly reinforced training (on 100% methyl anthranilate).

#### Time of injection

Injections of both agonists and antagonists were made at times between 5 min before and 40 min after training to determine the times at which memory consolidation could be impaired or enhanced. Retention was tested 120 min after training.

#### Time of test

With a time of injection determined, several groups of chicks were injected with the agonist or antagonist and memory retention tested in separate groups of chicks at discrete time intervals after training to identify the memory stage at which the drug effect became apparent.

#### Specificity of drug/receptor interaction

To determine subtype-specificity of 5-HT’s memory-enhancing effect after weakly reinforced learning, its ability to enhance memory was tested in the presence of the more or less selective antagonists (SB2212384 or methiothepin). The antagonist was administered systemically 5 min before the agonist was given 20 min after training. Subtype-specificity was indicated by the requirement of a higher dose of the agonist to enhance memory in the presence of the antagonist.

#### DAB challenges of memory enhancement of weak training by 5-HT and fluoxetine

To determine whether glycogenolysis was involved in the enhancement of memory by 5-HT agonists, the inhibitor of glycogenolysis, DAB, was injected subcutaneously 5 min before weak training with the agonist given 2.5 min after training, or 15 min before agonist injection at 20 min.

## RESULTS

### DOSE-RESPONSE FUNCTION FOR 5-HT WITH WEAKLY- AND STRONGLY-REINFORCED TRAINING

**FIGURE 1:**
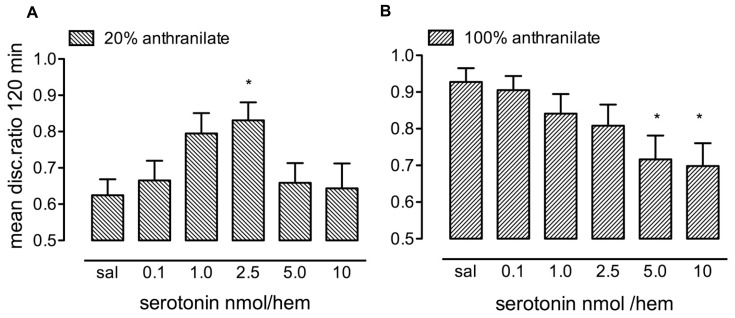
**Dose-response relationship for serotonin injected immediately after training. (A)** After training on 20% anthranilate there was significant enhancement of consolidation with a dose of 2.5 nmol/hem. **(B)** After strongly reinforced training on 100% anthranilate, doses of 5.0 and 10 nmol resulted in memory loss, *n* = 14–20 per group, *P < 0.05 compared to saline.

Ten μl of five different concentrations of 5-HT from 0.1 to 10 nmol per hemisphere (nmol/hem) were injected bilaterally into the IMM within 10 s after training on 20% anthranilate (**Figure 1A**) or 100% anthranilate (**Figure 1B**). Control chicks were injected with saline. Retention was tested at 120 min after training.

After training with the low anthranilate concentration, the saline control chicks showed very little retention of the aversive nature of the red bead at 120 min, with almost the same number of pecks on the red bead as on the blue bead, giving a mean DR of 0.625. There was a significant effect of serotonin dose [*F*_(5,101)_ = 2.68, *P* = 0.026] with retention levels in chicks injected with 2.5 nmol/hem being significantly greater than saline (*P* < 0.05). Higher doses of serotonin did not promote consolidation. The maximum DR obtained did not quite reach that of chicks trained on 100% anthranilate.

When administered after strongly reinforced training on 100% anthranilate (**Figure 1B**) the higher doses of serotonin (5 and 10 nmol/hem), which did not enhance memory consolidation with weakly reinforced training, now inhibited memory [*F*_(5,98)_ = 2.90, *P* = 0.017; 2.5 and 5 nmol/hem, *P* < 0.05].

### EFFECTIVE TIME OF ADMINISTRATION AND RETENTION FUNCTION

#### Weakly reinforced training

**FIGURE 2:**
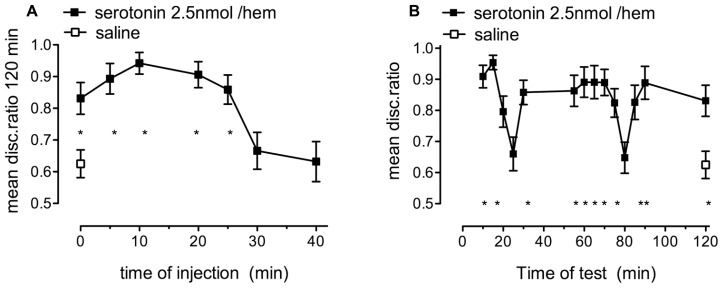
**The effect of serotonin on memory retention in chicks given weakly reinforced training. (A)** 2.5 nmol/hem dose of serotonin promoted the consolidation of memory when injected between +0 and 25 min after training (filled squares), whereas saline did not [(open square) *n* = 14–20 per group]. **(B)** When the same dose of serotonin was injected immediately after training memory was intact or almost intact after 120 min but the short-lasting amnestic periods, which normally occur at 15 and 55 min were delayed (filled squares), *n* = 14–20 per group, *P < 0.05 compared to saline-injected animals.

Separate groups of chicks trained with 20% anthranilate injected with 2.5 nmol/hem serotonin or saline immediately after training were tested at different intervals (**Figure 2A**). The saline injected controls did not remember at 120 min, whereas memory was intact in those treated with serotonin at any time during the first 25 min [*F*_(7,138)_ = 7.18, *P* < 0.001], but not thereafter. Serotonin had a greater effect when injected 10 min after training and this suggests that the dose-response relationship seen in **Figure 1** could have been more pronounced had this time of injection been used.

Normally, after training with 100% anthranilate and no drug-treatment, chicks tested for retention at selected times between 5 and 120 min, show dips in discrimination memory at 15 and at 55 min (Gibbs and Ng, 1979). At these two times the DRs falls to ~0.6. A quantitatively similar decline occurred when weakly reinforced training was followed by administration of serotonin (at +0 min) but with the dips in retention being delayed to 20–25 and 80 min, respectively (**Figure 2B**). This is similar to what occurs with noradrenaline and other agents that facilitate weakly reinforced memory (Gibbs and Summers, 2002). All DRs, apart from those tested at 20, 25, and 80 min, were significantly different from that after saline [*F*_(14,251)_ = 4.752, *P* < 0.001].

#### Strongly reinforced training

**FIGURE 3:**
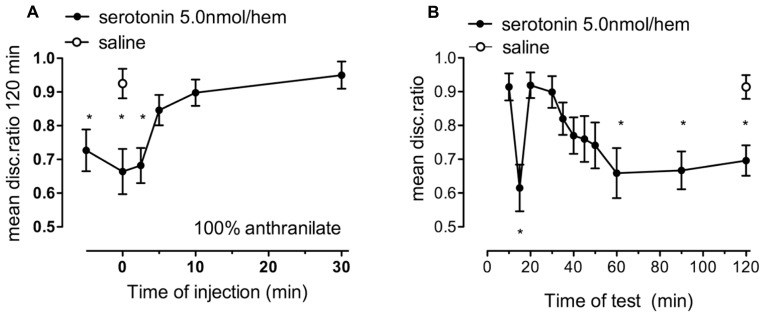
**The effect of serotonin on memory retention in chicks given strongly reinforced training. (A)** 5.0 nmol/hem serotonin resulted in the inhibition of memory consolidation when injected between -5 and +2.5 min relative to training, *n* = 16–19 per group. **(B)** this dose of serotonin injected immediately after training resulted in the gradual loss of memory after 30 min post training, *n* = 15–20 per group, *P < 0.05 compared to saline-injected animals.

As seen in the dose-response data, inhibition of memory occurred with higher doses of serotonin. Injection of 5 nmol/hem at different times after strongly reinforced training, led to amnesia when injections were made between 5 min before and 2.5 min after training (**Figure 3A**). One-way ANOVA revealed a significant effect of times of injection close to training [*F*_(6,115)_ = 5.67, *P* < 0.001] with -5, +0, and 2.5 min, being significantly different (*P* < 0.05) from saline (+0 min).

Chicks injected with 5 nmol/hem of serotonin showed good retention levels at tests, except for the usual 15 min dip, until 30 min after training. Thereafter, the discrimination started to decline, with amnesia fully developed by 60 min after training (**Figure 3B**) and DRs 15, 60, 90, and 120 min being different from those of saline injected (+0 min) chicks (*P* < 0.05).

### EFFECT OF THE BROAD-SPECTRUM 5-HT RECEPTOR ANTAGONIST METHIOTHEPIN

#### Dose-response function on strongly reinforced memory

**FIGURE 4:**
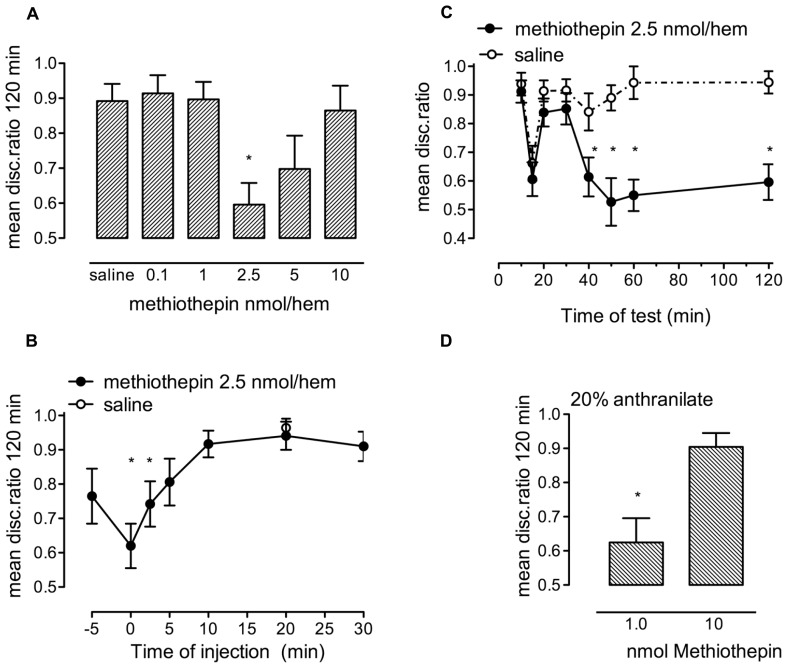
**Effect of the serotonin antagonist, methiothepin, on strongly reinforced memory. (A)** dose-response relationship for methiothepin injected immediately after training, *n* = 11–17 per group. **(B)** Time of injection of 2.5 nmol/hem of methiothepin relative to training and tested 120 min after training, *n* = 11–19 per group. **(C)** Memory retention in different groups of chicks tested at selected times after training and injection of methiothepin, *n* = 13–18 per group. *P < 0.05. **(D)** Test of two contrasting doses of methiothepin on memory following 20% anthranilate training, the low dose does not promote consolidation, whereas the discrimination ratio after administration of the high dose indicates normal memory retention, *n* = 16–18 per group. * statistically significant difference (*P* < 0.05) from saline-injected animals.

When methiothepin, which inhibits five of the seven 5-HT receptor families, including the 5-HT_1_ and 5-HT_2_ families, was injected immediately after strongly reinforced training (**Figure 4A**) there was an effect of drug concentration [*F*_(5,77)_ = 4.67, *P* = 0.001]. However, only the 2.5 nmol/hem dose had a significant inhibitory effect (*P* = 0.002).

#### Time of administration and retention function on strongly reinforced memory

Methiothepin impaired 120 min memory only when the inhibitor was injected immediately or 2.5 min after training [*F*_(7,112)_ = 4.57, *P* < 0.001), although there still appeared to be some inhibition when it was injected 5 min before or 5 min after training (**Figure 4B**). This is reminiscent of the inhibitory effect of high concentration of serotonin (**Figure 3A**). As with the high dose of serotonin, the methiothepin-induced memory deficit was significantly different from saline at 40 min post-training and all subsequent times [in contrast to most other statistical analyses this result was reached by two-way ANOVA drug x times *F*_(7,238)_ = 3.48, *P* < 0.05; **Figure 4C**].

#### Weakly reinforced memory

Although the higher dose of methiothepin (10 nmol/hem) did not inhibit strongly reinforced training (**Figure 4A**), it did consolidate weakly reinforced training (**Figure 4D**). 1.0 nmol/hem, which also had no effect on strong training (**Figure 4A**) did, however, not affect weakly reinforced training (**Figure 4D**). This result suggests that 10 nmol/hem rather than not having an effect on memory retention, could be facilitating promotion of poor memory. Together these observations suggest (i) that methiothepin acts on more than one 5-HT receptor involved in memory consolidation; (ii) that one of these two receptors promotes memory and the other inhibits it; and (iii) that methiothepin has different inhibitory potency on these receptors. For these reasons we turned to the more specific 5-HT_2B/C_ inhibitor SB221284.

#### Effect of selective 5-HT_2B/C_ receptor antagonist, SB221284

**FIGURE 5:**
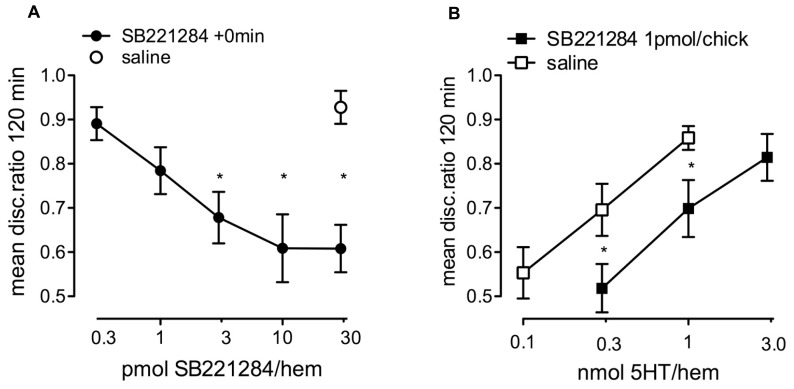
**Effect of the selective 5-HT_2B/C_ antagonist SB221284. (A)** Dose-response for SB221284 injected immediately after strongly reinforced training into the IMM with chicks tested 120 min after training *n* = 16–19 per group. **(B)** Challenge of 5-HT consolidation of weakly reinforced learning by prior injection of a low subcutaneous dose of SB221284 15 min after and administration of 5-HT into the IMM 20 min after training. The low dose of SB221284, that by itself did not inhibit memory, resulted in the need for a higher dose of 5-HT to enhance memory, *P* < 0.05, *n* = 15–16 per group. * statistically different (*P* < 0.05) in **A** from saline and in **B** from similar results in saline-injected animals.

Since a 10-fold higher concentration of serotonin was required to inhibit strongly reinforced learning than was needed to consolidate weakly reinforced learning (**Figure 1**), we hypothesized that the memory-enhancing effect of serotonin was exerted on a methiothepin-inhibitable 5-HT receptor with high affinity for serotonin, i.e., the 5-HT_2_, the 5-HT_6_, or the 5-HT_7_ receptor (Misane and Ogren, 2000). This idea was supported by the dose-response curve for the selective 5-HT_2B/C_ receptor antagonist SB221284, which abolished memory with 3 or 10 pmol/hem (**Figure 5A**; *F*_(5,93)_ = 6.99, *P* < 0.001), whereas 0.3 or 1.0 pmol/hem had no effect.

Specificity of action of 5-HT on 5-HT_2B/C_ receptors in memory enhancement was further demonstrated by challenging the 5-HT enhancement of consolidation of weakly reinforced learning by prior subcutaneous administration of 1 pmol/chick of SB221284. This low dose produced a significant shift in the dose-response function for 5-HT, demonstrating the requirement of a higher dose of 5-HT to enhance memory in the presence of SB221284 (**Figure 5B**). As in **Figure 4C** a two-way ANOVA was used to reveal a drug effect [*F*_(1,59)_ = 10.13, *P* = 0.002] and a significant dose effect [*F*_(1,59)_ = 10.50, *P* = 0.002].

#### Time of administration and retention function for SB221284

**FIGURE 6:**
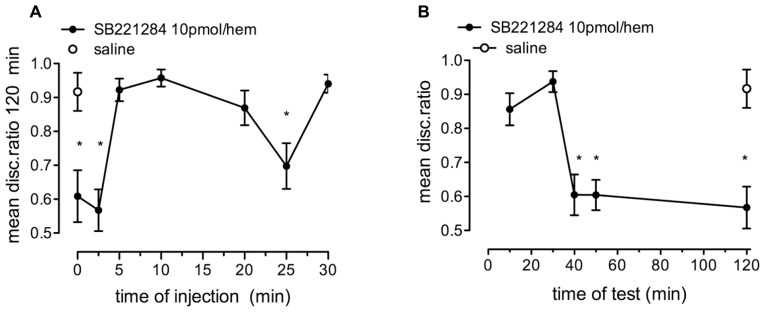
**Effect of the selective 5-HT_2B/C_ receptor antagonist, SB221284, on strongly reinforced memory. (A)** Time of injection of 10 pmol/hem of SB221284 relative to training and tested 120 min after training, *n* = 14–16 per group. **(B)** Memory retention tested at selected times after training and injection of 10 pmol SB221284 per hem 2.5 min after training, *n* = 14–16 per group, *P < 0.05 from saline-injected animals.

SB221284 (10 pmol/hem) resulted in significant memory loss after strongly reinforced training when injected immediately or 2.5 min after training, and again when it was injected 25 min after training. [**Figure 6A**; *F*_(7,114)_ = 9.07, *p* < 0.001]. Injection 5–20 min or 30 min after training did not affect memory processing.

When SB221284 (10 pmol/hem) was injected 2.5 min after strongly reinforced training, memory was intact on test 10 and 30 min after training but significantly reduced from 30 min on [**Figure 6B**; *F*_(5,85)_ = 11.60, *P* < 0.001].

### EFFECT OF FLUOXETINE AND PAROXETINE

**FIGURE 7:**
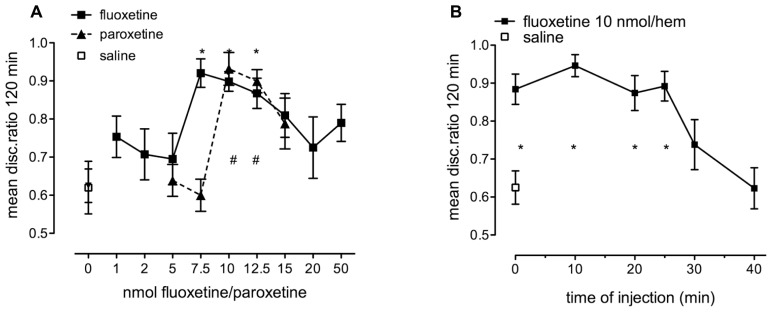
**(A)** Dose-response to fluoxetine (1–50 nmol/hem) and paroxetine (5.0–15 nmol/hem) administered into the IMM immediately after training on weakly reinforced learning, *n* = 11–20 per group. **(B)** Time of injection function after weakly reinforced training with injection of fluoxetine (10 nmol/hem) into the IMM at times from immediately after training to 40 min, *n* = 17–19 per group, *P < 0.05 from saline-injected animals.

The 5-HT_2B_- and astrocyte-specific 5-HT receptor agonists fluoxetine and paroxetine enhanced weakly reinforced training, with similar time course to 5-HT, but did not inhibit strongly reinforced training like serotonin (see **Figures 3A,B**). Both fluoxetine and paroxetine promoted consolidation in a dose-dependent manner (**Figure 7A**). The dose effects of both fluoxetine [*F*_(9,165)_ = 3.117, *P* = 0.002] and paroxetine [*F*_(5,92)_ = 8.882, *P* < 0.001] injected immediately after training were significant. As can be seen in **Figure 7A**, 10 nmol/hem of fluoxetine and paroxetine consolidate weakly reinforced learning at least equally as well as 2.5 nmol serotonin/hem, and there is at most a very small difference in potency between the two SSRIs. As with serotonin, the window for statistically significant memory consolidation by fluoxetine stretches from zero to 25 min after training [**Figure 7B**; *F*_(6,119)_ = 7.75, *P* < 0.001], with no effect at 30 and 40 min.

There was little inhibitory response found to fluoxetine at a high dose in sharp contrast to serotonin itself.

### EFFECT OF INHIBITION OF GLYCOGENOLYSIS BY DAB IN ENHANCEMENT BY 5-HT AND FLUOXETINE

**FIGURE 8:**
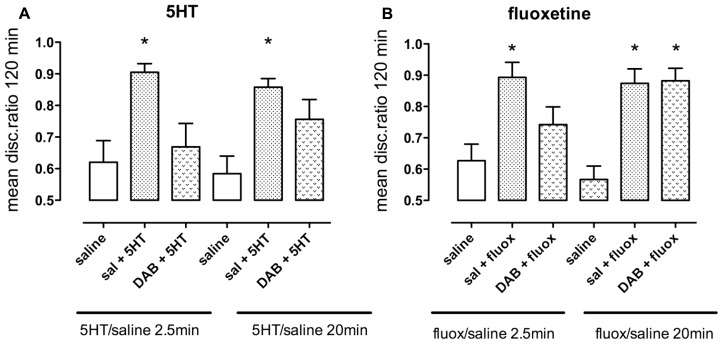
**Ability of the inhibitor of glycogenolysis, DAB, to prevent memory enhancement. (A)** Enhancement of weakly reinforced training by serotonin (1.0 nmol/hem, 2.5 min after training) was prevented by the sub-optimal dose of DAB (10 pmol/hem) given 5 min before training but not when DAB was given 15 min after training and serotonin 20 min after training. **(B)** Enhancement of weakly reinforced training by fluoxetine was only prevented by DAB at the early period and not the later (intermediate) one at 20 min, *n* = 12–20 per group, *P < 0.05 from animals injected with saline at the same time.

5-HT promotes weakly reinforced training when administered during both short-term and intermediate memory, i.e., administration 2.5 or 20 min after training (**Figure 8A**). A non-optimal dose of DAB (10 pmol/chick) prevented 5-HT (1 nmol/hem) from promoting weakly reinforced training during short-term [*F*_(2,47)_ = 6.20, *P* = 0.004] but not when given during intermediate memory.

Similarly, DAB prevented fluoxetine (7.5 nmol/hem) from promoting weakly reinforced training during short-term memory (fluoxetine added at 2.5 min; *F*_(2,49)_ = 6.80, *P* = 0.002), but not during intermediate memory (fluoxetine added at 20 min; **Figure 8B**).

## DISCUSSION

Serotonin exerts mnemonic actions on at least two different 5-HT receptors during discriminated learning in day-old chick. This is indicated by its production of a biphasic effect on memory with low doses facilitating weakly reinforced learning and higher doses inhibiting strongly reinforced learning. The two most important experimental observations in the present study are: (i) that the memory-enhancing effect of serotonin on weakly reinforced learning was at least as pronounced with any of the specific 5-HT_2B_ agonists fluoxetine or paroxetine, and that these two drugs were virtually equipotent; and (ii) that methiothepin or SB221284 needed to be administered almost immediately after strongly reinforced training to inhibit memory consolidation into long-term memory. Nevertheless, the inhibitory effect did not become manifest until after 30 min.

The first observation positively identifies the memory-enhancing effect of serotonin as being 5-HT_2B_-mediated, which is consistent with the effects of the inhibitory drugs. Although fluoxetine is not generally known to be a subtype-specific 5-HT_2B_ receptor agonist, this effect has been clearly documented in primary cultures of mouse astrocytes (Li et al., 2008). Paroxetine exerts similar and equipotent effects (Zhang et al., 2010). Qualitatitively and quantitatively identical effects were also found in intact mouse brain and brain slices (Li et al., 2008), and action of fluoxetine as a 5-HT_2B_ receptor agonist was confirmed *in vivo* (Diaz et al., 2012). Furthermore, administration of fluoxetine to mice for 2 weeks followed by fluorescence-activated cell sorting (FACS; Lovatt et al., 2007; Cahoy et al., 2008) has shown an astrocyte-specific increase in mRNA expression of the 5-HT_2B_ receptor, but of no other 5-HT_2_ receptors, as well as of the expression of several non-serotonergic genes (Li et al., 2012; Hertz et al., in this Research Project).

The effect of the glycogenolysis inhibitor, DAB, demonstrates that serotonergic 5-HT_2B_ receptor stimulation is the trigger of the glycogenolysis occurring soon after one-trial aversive learning in the day-old chick. As pointed out in Introduction, equipotent stimulation of this subtype of the 5-HT_2_ receptor by any of the conventional SSRIs stimulates a pathway leading to glycogenolysis (Kong et al., 2002; Hertz et al., 2012). During learning in day-old chicks DAB-mediated inhibition of the glycogenolytic period soon after training abolishes memory (Gibbs et al., 2006a). A normally occurring rise in IMM contents of glutamate, glutamine and aspartate in the left hemisphere simultaneous with the fall in glycogen (Hertz et al., 2003) is also inhibited by DAB (Gibbs et al., 2007). During the first 30 min post-training, two periods of glutamate release occur in the forebrain (Daisley et al., 1998). One period occurs almost immediately after the aversive experience, when glutamate release is confined to the left hemisphere. A second release, 30 min later, appears to occur bilaterally (Daisley and Rose, 1994), with preponderance of the right hemisphere, when glutamate level is again increased (Hertz et al., 2003), although glycogen content does not decrease. Increased pool sizes of glutamate probably reflect increased astrocytic synthesis of glutamate and its transfer via glutamine (a non-excitotoxic glutamate product and precursor) to neurons, where it is converted back to glutamate and used as transmitter glutamate (Hertz et al., 2003). Behavioral evidence supports the idea that DAB administration exerts its amnestic effect by preventing glutamate supply, since the extinction of memory following DAB administration 5 min before training can be counteracted by injection of glutamine at the time of training. Injection of lactate or the astrocyte-specific substrate acetate combined with aspartate (compensating for the inability of acetate [in contrast to lactate] to sustain the pyruvate carboxylation necessary for glutamate formation) has a similar effect (Gibbs et al., 2008a). These compounds rescue memory when injected either at training or 10 min before the second glycogenolytic period, but they are not effective later than 20 min after training (Gibbs et al., 2008a). The effectiveness of acetate demonstrates that the effect is exerted on astrocytes.

In addition to the inhibition in learning that occurs after 30 min when either methiothepin or the more selective 5-HT_2B/C_ inhibitor SB221284 is injected close to training, a dip in the DR occurs after injection at 25 min (see **Figure 6A**) suggesting that serotonin may also partly contribute to the second period of sensitivity to DAB around 30 min, when no glycogenolysis is apparent (O’Dowd et al., 1994; Gibbs et al., 2006b). However, more complete inhibition of memory (continuation beyond 30 min) by propranolol indicates that noradrenaline release and β_2_-adrenergic stimulation is the most important trigger of glycogenolysis at this time. The serotonergic system, in conjunction with the noradrenergic, dopaminergic and cholinergic systems, operates as part of the diffuse modulatory system of the central nervous system (e.g., Myhrer, 2003). All are implicated in learning and memory. Serotonin is often involved in emotionally tainted events, whereas the noradrenergic system shows a less specific modus of activation, but due to its widespread presence interacts with many other transmitter systems, in this case serotonin.

Simultaneous noradrenergic stimulation at α_2_- and β_3_-adrenergic receptors may explain the lack of an obvious decrease in glycogen content 30 min after training. This is because α_2_-adrenergic receptor stimulation promotes glycogen synthesis (Hertz et al., 2007; Hutchinson et al., 2011), which occurs simultaneously with glycogenolysis, as demonstrated by the memory inhibition resulting from injection of an α_2_-adrenergic antagonist up to 30 min post-training (Hertz and Gibbs, 2009). The importance of interaction between glycogenolysis and glycogen synthesis has also been demonstrated by Gibbs and Hutchinson (2012). Moreover, noradrenergic stimulation of β_3_-adrenergic receptors stimulates glucose uptake up to 25 min after training (Gibbs and Summers, 2000; Gibbs et al., 2008b). In contrast, activation of serotonergic receptors does not stimulate glycogen synthesis or glucose uptake in brain.

The lack of inhibition by the high dose of methiothepin could be due to inhibition of autoreceptors, probably 5-HT_1A_ receptors, at this dose level, resulting in increased extracellular levels of serotonin and the enhancement of memory consolidation. The result in **Figure 4D** that 10 nmol/hem did consolidate weakly reinforced training, observed after 120 min, whereas 1.0 nmol/hem had no effect on weakly reinforced training is consistent with the concept that the higher doses acting on autoreceptors, increased extracellular 5-HT levels and therefore produce enhancement of weak learning.

Other authors have also demonstrated the importance of glycogenolysis in learning. Suzuki et al. (2011) showed that memory and long-term potentiation (LTP) were abolished by glycogenolytic inhibitor administration in rat hippocampus. Newman et al. (2011) confirmed memory impairment by inhibition of intrahippocampal glycogenolysis. This impairment could be counteracted by either lactate or glucose. Since blockade of the neuronal monocarboxylate transporter impaired memory they concluded that lactate might be a *specially important substrate* for neurons during working memory, acting by rapidly providing additional energy. However, the ability of neurons to metabolize glucose- or lactate-derived pyruvate may not have been intact in the presence of the mononocarboxylate transport inhibitor (Halestrap and Denton, 1975). Also, at least in cultured neurons, lactate is not capable of supporting glutamatergic activity (Bak et al., 2006, 2012), which is so essential for learning (Gibbs and Hertz, 2005). Neuronal lactate utilization cannot be the mechanism operating during one-trial aversive learning in day-old chicks, since the astrocyte-specific substrate acetate in combination with aspartate (which does not provide a rapidly metabolizeable substrate to neurons) rescued memory in these animals after DAB administration. The importance of glycogen for learning has been substantiated by Duran et al. (2013), who demonstrated severe disturbances in long-term memory formation *and* learning-dependent synaptic plasticity in mice lacking brain glycogen synthase.

In their introduction of the Suzuki et al. (2011) paper in “Neuron,” Bezzi and Volterra (2011) suggested that aspects like the trigger(s) and timing(s) of astrocytic glycogenolysis during learning need further investigation. We had previously shown that glycogenolysis is essential for production of transmitter glutamate during one-trial aversive learning in the day-old chick and occurs during three periods during the first hr after training (Gibbs et al., 2008a). It has also long been known that noradrenaline triggers the second period of glycogenolysis and we now present evidence that the first period is triggered by serotonin acting on 5-HT_2B_ receptors. The trigger for the third period remains unknown.

## Conflict of Interest Statement

The authors declare that the research was conducted in the absence of any commercial or financial relationships that could be construed as a potential conflict of interest.
